# Supplementary material for: FAM134B-RHD Protein Clustering Drives Spontaneous Budding of Asymmetric Membranes
Source: J Phys Chem Lett. 2021 Feb 16;12(7):1926–31. doi: 10.1021/acs.jpclett.1c00031 (PMC8028312; doi:10.1021/acs.jpclett.1c00031)
Supplement: Supplementary file 2 — jz1c00031_si_002.pdf [file jz1c00031_si_002.pdf]

Supporting Information: FAM134B-RHD Protein Clustering Drives Spontaneous Budding of Asymmetric Membranes

Marc Siggel,[†] Ramachandra M. Bhaskara,[†,‡,§] Melanie K. Moesser,[†] Ivan Đikić,[‡,¶,§] and Gerhard Hummer[*,†,‖]

†*Department of Theoretical Biophysics, Max Planck Institute of Biophysics, Max-von-Laue Str. 3, 60438 Frankfurt am Main, Germany*

‡*Institute of Biochemistry II, Faculty of Medicine, Goethe University Frankfurt, Theodor-Stern-Kai 7, 60590 Frankfurt am Main, Germany*

¶*Max Planck Institute of Biophysics, 60438 Frankfurt am Main, Germany*

§*Buchmann Institute of Molecular Life Sciences, Goethe University Frankfurt, Max-von-Laue Straße 15, 60438 Frankfurt am Main, Germany*

‖*Institute of Biophysics, Goethe University Frankfurt, 60438 Frankfurt am Main, Germany*

E-mail: gerhard.hummer@biophys.mpg.de

This document contains

- Extended Methods
- Supporting Table and Figures
- Supporting Movie Legend

Extended Methods

System setup and MD Simulations. We used the MARTINI[1] coarse-grained model for the MD simulations. Coarse-grained 1-palmitoyl-2-oleoyl-glycero-3-phosphocholine (POPC) bilayers with varying degrees of asymmetry ($\Delta N = 100, 200, 300, 400$) were first built using the *insane.py* script.[2] The bilayers were then solvated with coarse-grained water containing 150 mM NaCl. All protein structures (FAM134B, $KALP_{15}$, opsins) were coarse-grained using the *martinize.py* script. For FAM134B, we used the coarse-grained model with secondary structure restraints as previously reported.[3] Control simulations were performed with $KALP_{15}$ and opsin. The $KALP_{15}$ peptide was modeled as an alpha helix with secondary structure restraints. The coarse-grained opsin molecules were modeled based on a crystal structure (PDB ID: 4J4Q) and used an elastic network with the default cutoff of 0.9 nm[4] to maintain the tertiary structure. All proteins were embedded in the asymmetric membranes ($30 \times 30 \times 18$ nm^3) in a square grid and energy minimized using a soft-core potential and steepest-decent algorithm to remove steric-clashes with lipids.

MD simulations were then performed using GROMACS (v. 2018.7).[5] We used the MARTINI force field (v.2.2.),[1,6] a 20 fs time-step, and a pair-interaction cutoff of 1.1 nm. Electrostatic interactions were modeled using the Coulomb potential with a reaction field. A dielectric constant of 15 was used to model interactions beyond the cutoff distance $r_c = 1.1$ nm. Electrostatic interactions were shifted to zero between 0 and 1.1 nm. Short-range Lennard-Jones interactions were also cut off at a distance of 1.1 nm and shifted to zero between 0.9 to 1.1 nm. The default Verlet-buffer cutoff scheme was used. The system temperature and pressure were maintained at 1 bar and 310 K, respectively, by using the velocity-rescale thermostat[7] and the Parrinello-Rahman barostat.[8] The Berendsen barostat was used for equilibration.[9] Production runs were simulated for at least 2 μs for data collection and analysis in all cases. Table S1 lists all systems built and simulated.

Analysis of MD trajectories. The simulation trajectories and individual frames were visualized using VMD.[10] Analysis scripts using MDAnalysis (v0.19)[11,12] were implemented in python 3.7 and plots were generated using matplotlib (v.3.1),[13] SciPy (v1.2), and NumPy (v1.16).[14,15] Lipids corresponding to individual leaflets were analyzed using the LeafletFinder algorithm in MDAnalysis.[11] To analyze the segregation of the proteins into the emerging membrane bud, the entire system was translated such that the lowest point of the lower leaflet ($N_{\mathrm{upper}} > N_{\mathrm{lower}}$) was set to $z = 0$ nm and centered in the xy plane using the highest point of the membrane for each frame. By tracing the highest and lowest z-positions of the phosphate (PO4) beads, we were able to locate and measure the extent of budding from the flat part of the bilayer. We monitored the time-traces of the z-component of the protein centers of mass to ascertain their involvement in (i) curvature induction, (ii) cluster formation, (iii) nascent stages of budding and (iv) segregation to the bud.

Analysis of FAM134B clusters. We quantified the FAM134B interactions by clustering of the transmembrane helical hairpins described in ref 3. Each hairpin was treated as an individual entity to account for the dynamic nature of the inter-hairpin connection.[3] Two hairpins were considered to be part of the same cluster if their centers of mass were within a distance of 3.5 nm.

Protein-protein contact maps. Contact maps were generated for all protein pairs (X, Y) and all frames $t = 1, \ldots, T$ in a given data set. First, all inter-protein pair-wise backbone (BB beads) distances were calculated. A residue pair (i, j) was considered to be in contact ($q_{\mathrm{inter}} = 1$) if any pair of BB beads was within 1.5 nm, with $q_{\mathrm{inter}} = 0$ otherwise. The number of contacts was then averaged over frames and protein pairs according to

$$\langle q_{\mathrm{inter}}(i, j)\rangle = \frac{2}{n(n+1)T} \sum_{t=1}^{T} \sum_{X=1}^{n-1} \sum_{Y=X+1}^{n} q_{\mathrm{inter}}(i, j|X(t), Y(t)). \quad \text{(S1)}$$

Two data sets were analyzed: (i) all replicas at all concentrations for asymmetry $\Delta N = 300$ and (ii) all replicas at all asymmetries $\Delta N = 100$ to 400 with $n = 9$ with essentially the same results.

Table S1: MD simulations. Listed are the number of proteins n, the lipid number asymmetry ΔN, the number of lipids in the lower (N_{lower}) and upper leaflets (N_{upper}), the system dimensions $L_x \times L_y \times L_z$ at the start of the simulation, the simulation time, the number of replicate simulations of each setup, and the number of observed budding events (with bicelle formation in parentheses). The bottom block lists the simulations with other proteins and with FAM134B in reverse topology.

n	ΔN ($N_{\text{lower}}/N_{\text{upper}}$)	System size [nm^3]	Simulation time [μs]	Replicates	budding (bicelle)
1	100 (1421/1521)	30×30×18	>2	3	0
2	100 (1421/1521)	30×30×18	>2	3	0
3	100 (1421/1521)	30×30×18	>2	3	0
4	100 (1421/1521)	30×30×18	>2	3	0
5	100 (1421/1521)	30×30×18	>2	3	0
6	100 (1421/1521)	30×30×18	>2	3	0
7	100 (1421/1521)	30×30×18	>2	3	0
8	100 (1421/1521)	30×30×18	>2	3	0
9	100 (1421/1521)	30×30×18	>2	3	0
10	100 (1421/1521)	30×30×18	>2	3	0
11	100 (1421/1521)	30×30×18	>2	3	0
12	100 (1421/1521)	30×30×18	>2	3	0
13	100 (1421/1521)	30×30×18	>2	3	0
1	200 (1321/1521)	30×30×18	>2	3	0
2	200 (1321/1521)	30×30×18	>2	3	0
3	200 (1321/1521)	30×30×18	>2	3	0
4	200 (1321/1521)	30×30×18	>2	3	0
5	200 (1321/1521)	30×30×18	>2	3	0
6	200 (1321/1521)	30×30×18	>2	3	0
7	200 (1321/1521)	30×30×18	>2	3	0
8	200 (1321/1521)	30×30×18	>2	3	1
9	200 (1321/1521)	30×30×18	>2	3	3
10	200 (1321/1521)	30×30×18	>2	3	2
11	200 (1321/1521)	30×30×18	>2	3	2
12	200 (1321/1521)	30×30×18	>2	3	2
13	200 (1321/1521)	30×30×18	>2	3	2
1	300 (1221/1521)	30×30×18	>2	3	0
2	300 (1221/1521)	30×30×18	>2	3	0
3	300 (1221/1521)	30×30×18	>2	3	2
4	300 (1221/1521)	30×30×18	>2	3	2
5	300 (1221/1521)	30×30×18	>2	3	3
6	300 (1221/1521)	30×30×18	>2	3	3
7	300 (1221/1521)	30×30×18	>2	3	2
8	300 (1221/1521)	30×30×18	>2	3	3
9	300 (1221/1521)	30×30×18	>2	3	3
10	300 (1221/1521)	30×30×18	>2	3	3
11	300 (1221/1521)	30×30×18	>2	3	3
12	300 (1221/1521)	30×30×18	>2	3	3
13	300 (1221/1521)	30×30×18	>2	3	3
1	400 (1121/1521)	30×30×18	>2	3	1(2)
2	400 (1121/1521)	30×30×18	>2	3	1(2)
3	400 (1121/1521)	30×30×18	>2	3	0(3)
4	400 (1121/1521)	30×30×18	>2	3	2(1)
5	400 (1121/1521)	30×30×18	>2	3	3
6	400 (1121/1521)	30×30×18	>2	3	3
7	400 (1121/1521)	30×30×18	>2	3	2(1)
8	400 (1121/1521)	30×30×18	>2	3	2(1)
9	400 (1121/1521)	30×30×18	>2	3	3
10	400 (1121/1521)	30×30×18	>2	3	3
11	400 (1121/1521)	30×30×18	>2	3	3
12	400 (1121/1521)	30×30×18	>2	3	3
13	400 (1121/1521)	30×30×18	>2	3	3
9(opsins)	300 (1221/1521)	30×30×18	12	1	0
15(KALP$_{15}$	300 (1221/1521)	30×30×18	12	1	0
15(reverse)	300 (1221/1521)	30×30×18	12	1	0

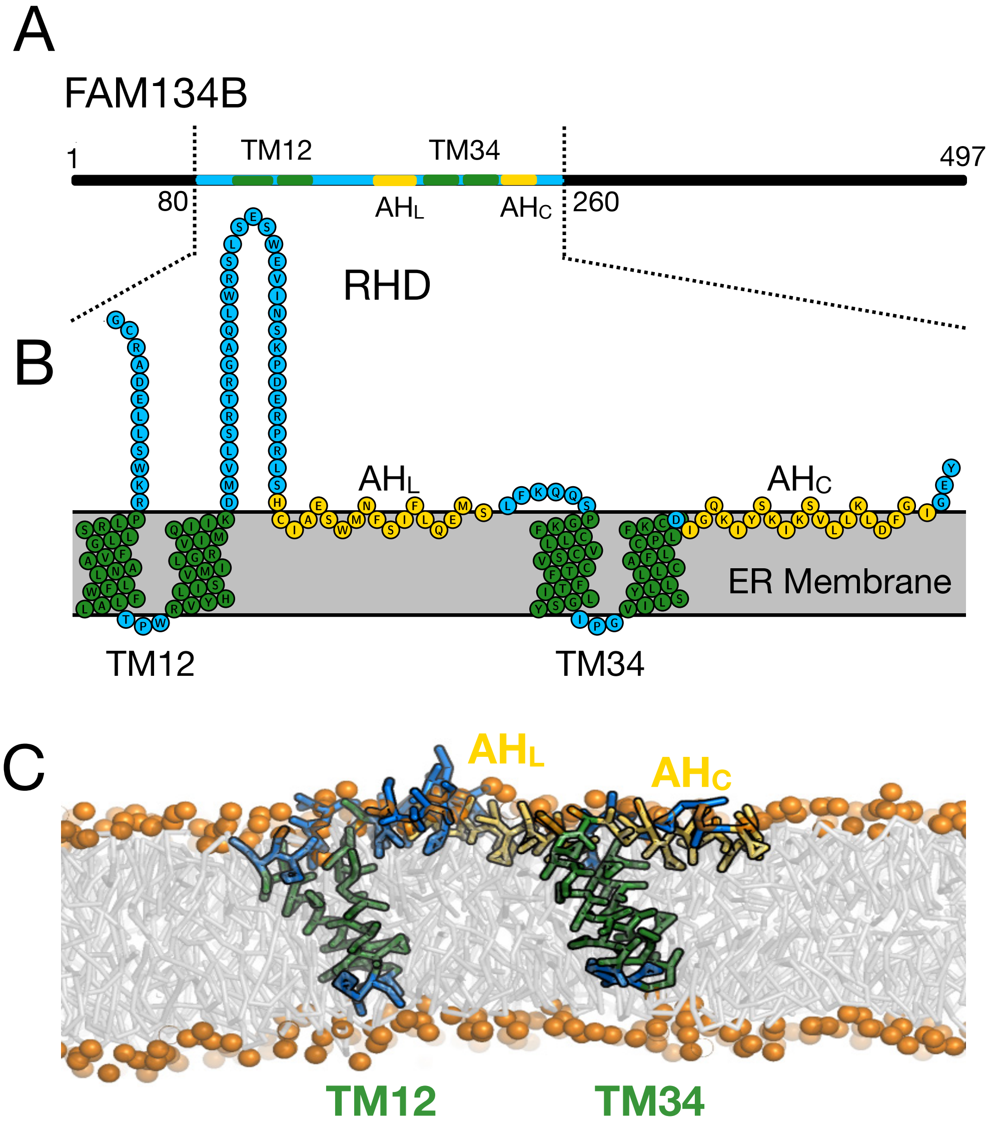

Figure S1: Sequence, topology and structure of FAM134B-RHD. (A) Schematic representation of the FAM134B sequence containing the RHD (80-260). The two transmembrane hairpins (green, TM12 and TM34), and the two conserved amphipathic helices (yellow, AH_L and AH_C) characterstic of the RHD are highlighted. Disordered regions are shown in blue. (B) Topology of FAM134B-RHD (80–260) indicating the organization of TM segments (green) and amphipathic helices (yellow) within the ER membrane (cytosol: top; lumen: bottom). (C) Snapshot of a coarse-grained MD simulation of FAM134B-RHD embedded in a POPC bilayer. Phosphate headgroups are shown in orange, lipid tails are shown as faint grey sticks. Structural features are colored as in (B).

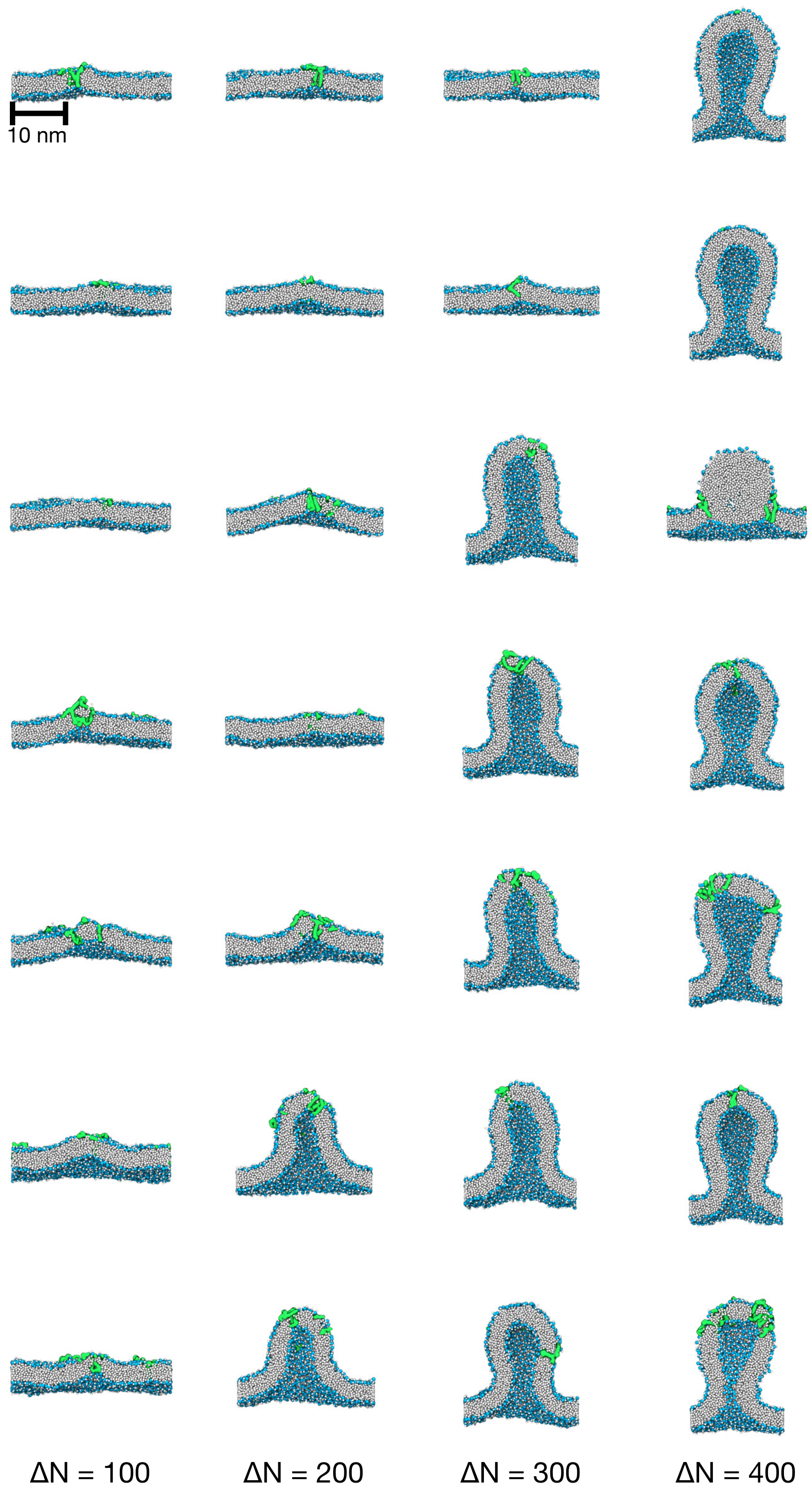

Figure S2: Structure and shape of membrane buds at low protein concentration. Snapshots show cross sections at the end of simulations with $n = 1, \ldots, 7$ RHD molecules (top to bottom) at asymmetries $\Delta N = 100, 200, 300, 400$ (left to right). The flat membrane or bicelle is shown for systems where no budding occurred. Phosphate groups are shown in light blue, and lipid tails in white. FAM134B-RHD is shown as green surface. Water and ions are omitted for clarity.

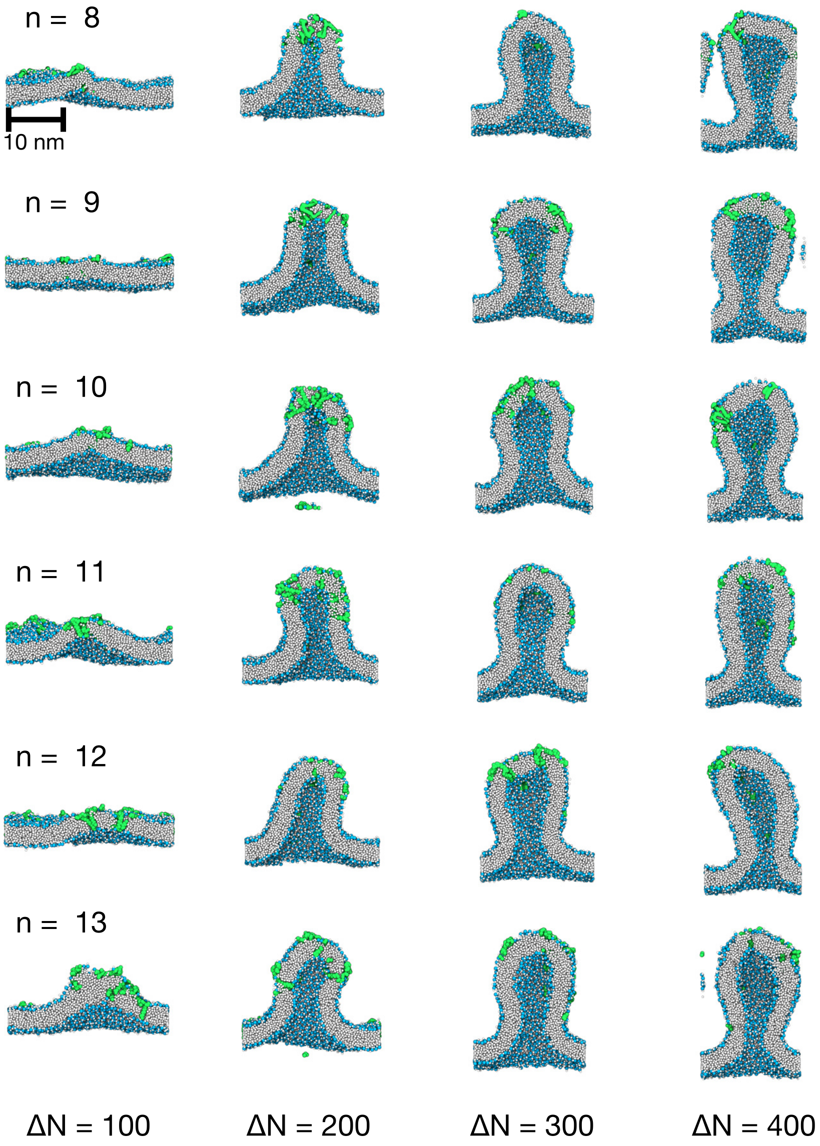

Figure S3: Structure and shape of membrane buds at high protein concentration. Snapshots show cross sections at the end of simulations with $n = 8, \ldots, 13$ RHD molecules (top to bottom) at asymmetries $\Delta N = 100, 200, 300, 400$ (left to right). The flat membrane or bicelle is shown for systems where no budding occurred. Phosphate groups are shown in light blue, and lipid tails in white. FAM134B-RHD is shown as green surface. Water and ions are omitted for clarity.

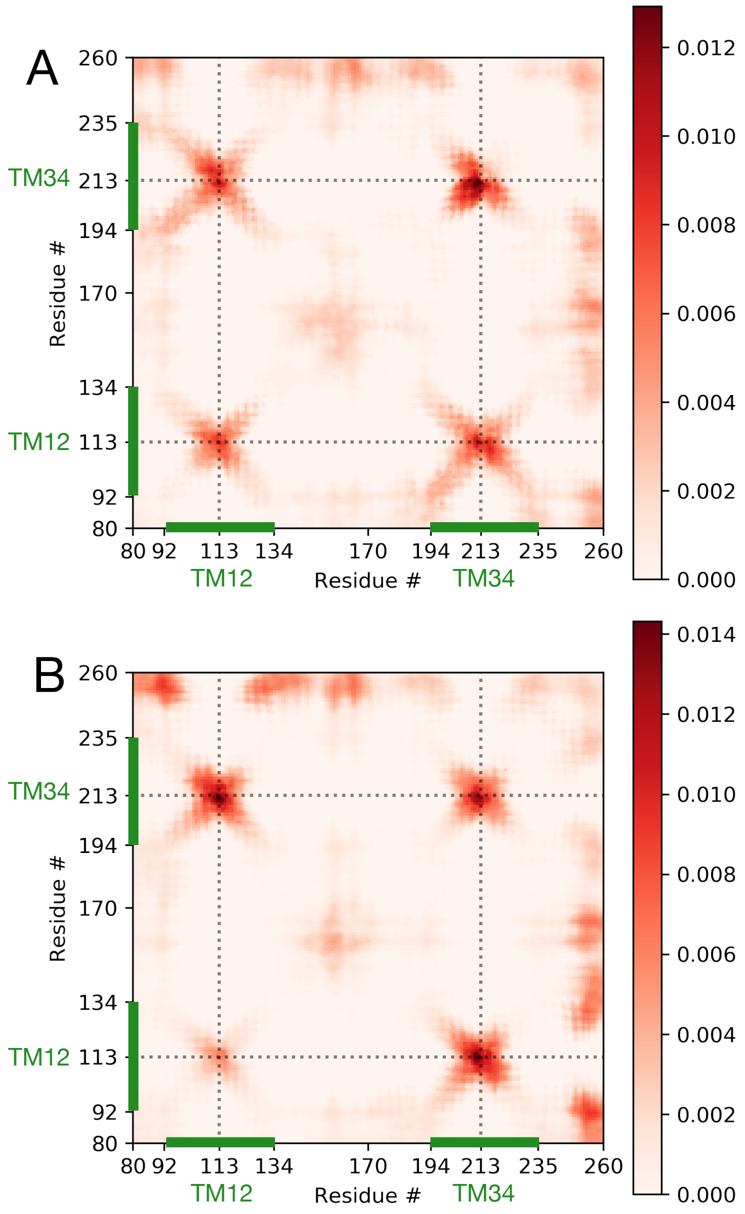

Figure S4: Protein-protein contact maps. Shown is the average number of contacts between distinct proteins resolved by residue-residue pairs as defined in eq S1. Green bars indicate TM12 and TM34, and dashed lines the intervening luminal loops. (A) Average over all replicas at all asymmetries $\Delta N = 100, 200, 300, 400$ with $n = 9$ FAM134B-RHDs. (B) Average over all replicas at $\Delta N = 300$ with $n = 2$ to 13 FAM134B-RHDs.

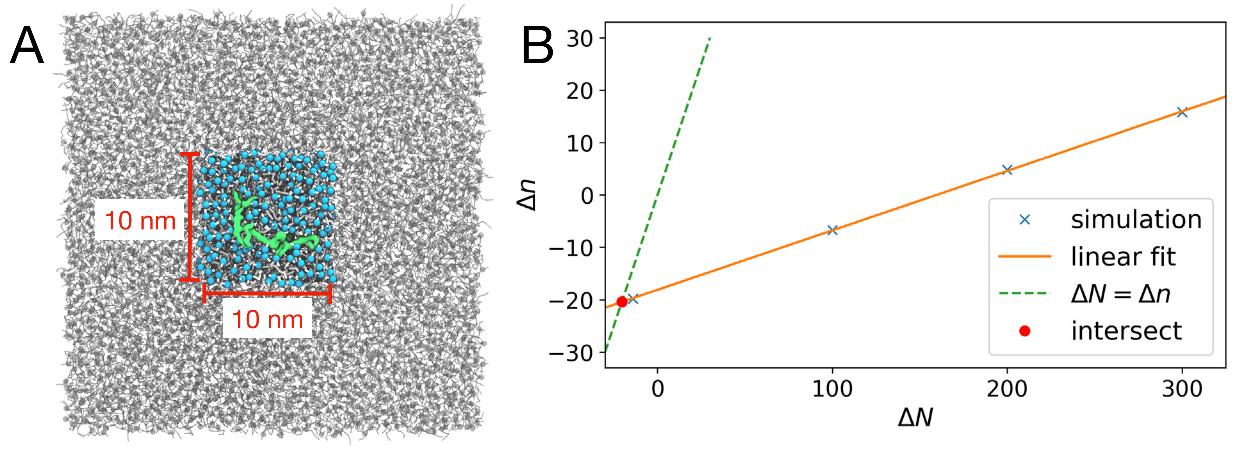

Figure S5: Asymmetric footprint of FAM134B in the membrane. (A) Top view of a system with a single FAM134B-RHD molecule in an asymmetric membrane ($\Delta N = 100$). The blue lipids indicate the 10×10 nm^2 square around the center of mass of FAM134B-RHD (green), within which lipid numbers n_{upper} and n_{lower} in the two leaflets were counted and averaged. (B) Difference $\Delta n = n_{\text{upper}} - n_{\text{lower}}$ in the average number of lipids within the square patch around FAM134B-RHD as a function of the leaflet asymmetry $\Delta N = N_{\text{upper}} - N_{\text{lower}}$ for simulations at $\Delta N = -14, 100, 200, 300$. A straight-line fit (orange) gives $\Delta n = -18.1 + 0.114\Delta N$. In a large membrane, we expect $\Delta n = \Delta N$ (dashed green line) on average, such that the lipid asymmetry of the membrane exactly compensates for the asymmetric footprint of the protein. From the intersection of $\Delta n = \Delta N$ with the linear fit, we find that at equilibrium, FAM134B-RHD displaces 20.4 lipids more from the upper leaflet than from the lower leaflet (red circle).

Movie Legends

Supporting Movie S1

MD simulation trajectory of membrane budding event induced by FAM134B-RHD. A simulation of nine FAM134B-RHD molecules embedded in a membrane with asymmetry $\Delta N = 300$ is shown. The movie covers a time of 2.5 μs. The same trajectory is analyzed in Figures 1, 3 and 5 of the main text. Phosphate groups are shown in light blue, and lipid tails in white. The proteins are colored in green, orange, yellow and grey, respectively (same coloring as in Figure 5 of the main text). In each frame, the trajectory was centered on the highest phosphate (PO4) bead in z-direction, as described in the Methods section.

References

(1) Marrink, S. J.; Risselada, H. J.; Yefimov, S.; Tieleman, D. P.; De Vries, A. H. The MARTINI Force Field: Coarse Grained Model for Biomolecular Simulations. *J. Phys. Chem. B* **2007**, *111*, 7812–7824.

(2) Wassenaar, T. A.; Ingólfsson, H. I.; Böckmann, R. A.; Tieleman, D. P.; Marrink, S. J. Computational Lipidomics With Insane : A Versatile Tool for Generating Custom Membranes for Molecular Simulations. *J. Chem. Theory Comput.* **2015**, *11*, 2144–2155.

(3) Bhaskara, R. M.; Grumati, P.; Garcia-Pardo, J.; Kalayil, S.; Covarrubias-Pinto, A.; Chen, W.; Kudryashev, M.; Dikic, I.; Hummer, G. Curvature Induction and Membrane Remodeling by FAM134B Reticulon Homology Domain Assist Selective ER-phagy. *Nat. Commun.* **2019**, *10*, 2370.

(4) Park, J. H.; Morizumi, T.; Li, Y.; Hong, J. E.; Pai, E. F.; Hofmann, K. P.; Choe, H. W.; Ernst, O. P. Opsin, a Structural Model for Olfactory Receptors? *Angew. Chemie - Int. Ed.* **2013**, *52*, 11021–11024.

(5) Abraham, M. J.; Murtola, T.; Schulz, R.; Páll, S.; Smith, J. C.; Hess, B.; Lindahl, E. GROMACS: High Performance Molecular Simulations Through Multi-Level Parallelism From Laptops to Supercomputers. *SoftwareX* **2015**, *1-2*, 19–25.

(6) Tieleman, D. P.; Marrink, S.-J.; Kandasamy, S. K.; Periole, X.; Monticelli, L.; Larson, R. G. The MARTINI Coarse-Grained Force Field: Extension to Proteins. *J. Chem. Theory Comput.* **2008**, *4*, 819–834.

(7) Bussi, G.; Donadio, D.; Parrinello, M. Canonical Sampling Through Velocity Rescaling. *J. Chem. Phys.* **2007**, *126*, 014101.

(8) Parrinello, M.; Rahman, A. Polymorphic Transitions in Single Crystals: A New Molecular Dynamics Method. *J. Appl. Phys.* **1981**, *52*, 7182–7190.

(9) Berendsen, H. J. C.; Postma, J. P. M.; van Gunsteren, W. F.; DiNola, A.; Haak, J. R. Molecular Dynamics With Coupling to an External Bath. *J. Chem. Phys.* **1984**, *81*, 3684–3690.

(10) Humphrey, W.; Dalke, A.; Schulten, K. VMD: Visual Molecular Dynamics. *J. Mol. Graph.* **1996**, *14*, 33–38.

(11) Michaud-Agrawal, N.; Denning, E. J.; Woolf, T. B.; Beckstein, O. MDAnalysis: A Toolkit for the Analysis of Molecular Dynamics Simulations. *J. Comput. Chem.* **2011**, *32*, 2319–2327.

(12) Gowers, R.; Linke, M.; Barnoud, J.; Reddy, T.; Melo, M.; Seyler, S.; Domański, J.; Dotson, D.; Buchoux, S.; Kenney, I.; Beckstein, O. MDAnalysis: A Python Package for the Rapid Analysis of Molecular Dynamics Simulations. Proc. 15th Python Sci. Conf. 2016.

(13) Hunter, J. D. Matplotlib: A 2D Graphics Environment. *Comput. Sci. Eng.* **2007**, *9*, 90–95.

(14) Oliphant, T. E. Python for Scientific Computing. *Comput. Sci. Eng.* **2007**, *9*, 10–20.

(15) Harris, C. R. et al. Array Programming With NumPy. *Nature* **2020**, *585*, 357–362.
